# Adapting a Systems-based Intervention to Improve Older Adult Patient Mobility in Hospital Settings

**DOI:** 10.1093/geroni/igaf122.2001

**Published:** 2025-12-31

**Authors:** Barbara King, David Tucker, Linsey Steege

**Affiliations:** University of Wisconsin–Madison, Madison, Wisconsin, United States; University of Wisconsin–Madison, Madison, Wisconsin, United States; University of Wisconsin–Madison School of Nursing, Madison, Wisconsin, United States

## Abstract

Hospitalized older adults often experience functional decline due to low mobility. Acute care nurses are responsible for maintaining patient functional status but often do not walk patients due to multiple system barriers. To address system barriers, we developed Mobilizing Older adults Via a systems-based INtervention (MOVIN) a multicomponent mobility model of care. However, implementing multicomponent mobility interventions in hospitals can be complex often requiring adaptations to meet unique needs of inpatient units. A cluster randomized controlled trial design was used paired with a participatory approach to implementing MOVIN. Four inpatient units across 2 hospitals served as study sites. Adaptations of MOVIN to meet the unique needs of each intervention unit were made. A Fidelity rating tool was used to evaluate adherence to the core components of MOVIN. An interrupted time series was used to evaluated changes in percent ambulation and ambulation distance across pre, intervention, and post intervention periods. Adaptations for MOVIN occurred in psychomotor training, mobility resources, and communication tools due to staff turnover, physical layout of the unit, and unique unit workflows. Adaptations occurred during the planning (proactive adjustment) as well as the active (reactive to ongoing barriers) intervention period. Fidelity of all MOVIN components was maintained with very good to excellent ratings. Unit ambulation outcomes (percent ambulation and ambulation distance) significantly increased across pre, intervention, and post-intervention periods and sustained for all four units. Further research is needed on navigating complexities in implementing multicomponent interventions in hospital settings to improve older adult patient mobility.

